# Discretionary Effort on Green Technology Innovation: How Chinese Enterprises Act when Facing Financing Constraints

**DOI:** 10.1371/journal.pone.0261589

**Published:** 2021-12-22

**Authors:** Kexian Zhang, Xiaoying Liu, Min Hong

**Affiliations:** School of Economics and Management, Hunan Institute of Technology, Hengyang, China; Szechenyi Istvan University: Szechenyi Istvan Egyetem, HUNGARY

## Abstract

Firm’s effort on Green technology innovation (hereafter, called G-innovation) is affected by financing constraints, and firm will make a discretionary choice according to its own situation, to achieve the maximization of self-interests. Based on the data of Chinese micro enterprises, firstly, we empirically analyze firms’ decision-making towards G-innovation when faced with financing constraints. It supports the view that financing constraints can hinder enterprise technological innovation. And we also make an explanation that the social benefits of green technology innovation are greater than personal benefits, which makes enterprises tend to reduce green technology innovation when facing financing constraints. Then we examine firms’ heterogonous behaviors under different internal attributes and external environments. The results reveal that: First, firms are reluctant to pay more efforts to G-innovation when faced with increased financing constraints. Second, firms with different attributes exhibit heterogeneous G-innovation. Political connections will change firms’ willingness to innovate, while the structure of property rights and the pollution degree will not. Third, firms under different external environment also exhibit heterogeneous G-innovation. When economic policy uncertainty increases, firms’ willingness to innovate weakens. The development of shadow banks fail to improve firm’s willingness to innovate.

## Introduction

Firms are the main body of pollutant emission, and G-innovation has become the key means to improve environmental quality, which plays an important role in pollution governance [1]. However, G-innovation is vulnerable to financing constraints. This is because, on the one hand, unlike fixed asset investment, R&D investment lacks collateral value and is suffered from serious information asymmetry, which makes it vulnerable to adverse selection and moral hazard [2], and finally result in innovation underinvestment in firms with financing constraints. On the other hand, financing constraints may also cause firms to terminate profitable R&D investment projects when their performance declines. Green technology innovation mainly refers to the technological improvements that save energy, prevent pollution, or enable waste recycling [3, 4], which is different from frugal innovation and financial innovation. Frugal innovations mainly refer to the innovation which minimize the use of material and financial resources in the complete value chain though focusing on core functionalities [5, 6]. And financial innovation is the act of creating and then popularizing new financial instruments as well as new financial technologies, institutions and markets. Financial innovation can play a critical role in driving green innovation [7]. For example, green crowdfunding is a new method for financing investment in sustainable business projects.

The theoretical literature presents two opposing views on firm’s discretionary effort on technology innovation with financing constraints increased. One view mainly focuses on "more money, more innovation" [8]. They suggest that financing constraints will inhibit firm’s effort on technology innovation [9–11]. Ayyagari, Demirgüç-Kunt [12, 13] also conclude that there is a negative correlation between financing constraints and technology innovation, and financing constraints may lead firms to give up R&D projects [14]. The other view focuses on "less money, better innovation”. They believe that financing constraints help enterprises to choose more innovative projects [15, 16]. By comparing the differences of innovation performance of new firms (fewer resources) with old firms (more resources), Katila and Chen [17] argue that the impact of resource scarcity can promote firms’ innovation performance, and new enterprises may show higher innovation rate than old enterprises. Mishina, Pollock [18] argue that the existence of financial slack may indicate that it is inefficient, indicating that the organization lacks the spirit of innovation. Even some scholars have also explained from the perspective of psychology that when provided with fewer resources for solving innovation problems, subjects are more innovative [19]. From the perspective of empirical analysis, many literatures have investigated the relationship between financial constraints and firm’s technology innovation, but few literatures have analyzed how firm act towards green technology innovation when faced with financial constraints. Compared with general technology innovation, green technology innovation is more public, whose private return rate is significantly lower than the social return rate.

For countries in different stages of economic development, financing constraints have different effects on G-innovation. Lots of views demonstrate the effects of financing constraints on R&D investment in developed countries.Brown, Martinsson [2] use the data of European enterprises and conclude that the availability of financing is essential for a firm’s R&D investment. Hall [20] and Hall and Lerner [21] also argue that financing constraints hinder the physical investment and R&D expenditure in OECD countries. Cao and Leung [22] use the survey data of small and medium-sized enterprises in Canada and argue that financing constraints can inhibit Canada’s investment and thus employment growth. Álvarez and Crespi [23] also argue that financing constraints impede Chile’s technological innovation. However, the innovative activities of emerging market economies have been less investigated so far. [24] examines the impact of financing constraints on R&D expenditure in India and argues that internal cash flow had a significant positive impact on R&D expenditure. Zhang and Zheng [25] use Chinese manufacturing firm data and conclude that there is a positive correlation between innovation investment and cash flow in non-state-owned enterprises but no such correlation in state-owned enterprises.

Enterprises with different attributes may exhibit heterogeneous discretionary efforts when faced with increased financing constraints. So far, there are few studies about the specific impacts of financing constraints on firms’ innovation output with different attributes. Most studies mainly extend the impact of financing constraints on physical investment to R&D investment. Also, there is some evidence suggest that financial factors have a severe impact on innovative activities in higher-tech sectors, for smaller enterprises [26], and non-state-owned enterprises [25].

The marginal contribution of this paper mainly lies in: First, we investigate corporate decision-making behavior towards G-innovation given financing constraints. Differ from developed countries, the capital markets in emerging market economies is immature [27], and vulnerable to financing constraints, which is more likely to lead to underinvestment in emerging market countries. Therefore assessing whether and the extent to which financing constraints affect G-innovation becomes crucial. Second, we investigate firms’ heterogeneous discretionary efforts based on their different internal attributes. As Jiang and Kim [28] point out, there are many differences in corporate governance between China and other developed countries. For example, there are many SOEs in China, and the state is their largest shareholder; Debt ratios in China are quite high compared to developed countries. Therefore, to better evaluate the effects of financing constraints on corporate G-innovation in China, we need to classify firms into sub-subsamples according to property structures, political connections and pollution levels, and reexamine the heterogeneous effects. Third, we need to study firms’ heterogeneous discretionary efforts under different external environment. Two important changes in external environment are worth considering, one of which is the economic policy uncertainty. Firms are very sensitive to the policy changes, so that when economic policy uncertainty increases, firms often implement corresponding emergency measures. It is noting that financial constrained firms exhibit different G-innovation behavior from non-financial constrained firms. The second change in the external environment is the expansion of shadow banking in recent years. On the one hand, shadow banking can supplement the traditional commercial banks and ease the financing constraints. On the other hand, shadow banking can replace traditional finance and increase financing constraints. But when the replacement effect exceeds the supplement effect, the expansion of shadow banking will increase financial constraints and aggravate their inhibitory effect on corporate G-innovation.

The rest of the paper is organized as follows. Section 2 is the econometric test on corporate G-innovation behavior under the background of financing constraints. Section 3 is about firm’s discretionary behavior towards G-innovation based on its internal attributes. Section 4 is about firm’s discretionary behavior towards G-innovation based on the different external environments. And section 5 concludes.

## Econometric test on corporate G-innovation behavior with financing constraints

### Model design

Generally, firms may raise their funds from internal financing and external financing. According to the MM theory, when in a perfect market, corporate investment decisions are not related to capital structure. However, due to the market information asymmetry [29], the capital structure will have an impact on firm’s investment activities. For innovative activities, capital’s intangible nature will make the financing cost in R&D investment higher than that in physical investment. Specifically, R&D investment has no collateral value [30, 31], This will make banks and other lenders reluctant to lend to enterprises to engage in R&D investment, and then make a lot of innovation investment shelved, especially those still in the research stage. There are also some empirical studies supporting the restrictive effect of financing constraints on enterprise innovation activities, for example,Brown, Fazzari [32] argue that in newly established firms the level of financing is significantly related to the innovative activities. Using Chinese non listed firm data from 2000 to 2007, Guariglia and Liu [33] conclude that the innovative activities of Chinese enterprises are limited by internal financing.Gorodnichenko and Schnitzer [13] develop a theoretical model to demonstrate financing constraints will limit corporate innovation under the assumption of the pecking order theory. Of course, few scholars believed that financing constraints had no impact on the innovation activities of enterprises. For example, Bhagat and Welch [34] believe that past operating cash flow was not related to the current level of R&D expenditure. Bond, Harhoff [35] indicate that cash flow is not vital for R&D investment of German or British enterprises. Considering that Chinese capital market is still immature, which falls behind the developed countries, and the problem of capital mismatch is still very serious [36], we believe that whether they are listed companies or not, the problem of financing constraints is serious. Therefore, we put forward our first hypothesis:

**Hypothesis 1.** *Under the background of financing constraints*, *enterprises will reduce their G-innovation level*.

Furthermore, when faced with financing constraints, firms with different attributes show heterogeneous innovation behavior. Specifically, first, there exist heterogeneities among enterprises with different property rights. In China, it is easier for state-owned enterprises to get the financing from state-owned commercial banks compared with non-state-own enterprises, which is explained as “political peck order” by Huang [37]. Therefore, we believe that non-state-owned enterprises are more vulnerable to financing constraints. Second, there also exist heterogeneities among enterprises with different political connections. Enterprises associated with central or local governments are more likely to obtain funds from state-owned commercial banks. Therefore, we believe that non-political enterprises are more vulnerable to financing constraints. Third, it is worth mentioning that the heterogeneity may also exist among enterprises with different pollution levels. In recent years, more and more attention has been paid to green finance, especially with the implementation of green credit guidelines, commercial banks began to issue green credit. Therefore, we believe that compared with non-heavy polluting enterprises, heavy polluting enterprises are more vulnerable to financing constraints. This leads to our second hypothesis:

**Hypothesis 2.** *When financing constraints increase*, *enterprises with different attributes show heterogeneous G-innovation behavior*.

In addition, due to the irreversibility of investment, policy uncertainty can lead to preventive delays of investment, which will inhibit firm’s physical investment [38]. Bhattacharya, Hsu [39] and Andrey [40] conclude that policy uncertainty will cause firms the problems how to adjust to the change of policies, thus hindering enterprise innovation. Gorodnichenko and Schnitzer [13] develop a model to demonstrate that the higher the external financing cost, the greater the inhibitory effect of financing constraints on corporate technology innovation. We assume that the increase of economic policy uncertainty will raise the external financing costs, and thus aggravate the inhibition of technological innovation. Therefore, we put forward our third hypothesis:

**Hypothesis 3.** *Economic policy uncertainty will aggravate the inhibitory effect of financing constraints on corporate G-innovation*.

As an informal financing form, the scale of shadow banking has been expanding in recent years, which plays an important role in the process of enterprise financing. On the one hand, shadow banking can supplement the traditional commercial banks and ease the financing constraints. This is mainly reflected in the fact that shadow banking is an off-balance sheet extension of commercial banks, which is the second-best choice for enterprises when they cannot obtain financing from formal channels and makes up for the lack of formal credit to a great extent [41]. On the other hand, shadow banking will replace traditional finance and increase financing constraints. When the return on financial assets is higher than in the real economy, firms will increase the proportion of investment in financial assets to pursue profit maximization [42]. Banks are also unwilling to lend, and enterprises invest their idle funds in financial assets, forming a crowding-out effect on industrial investment [43]. Even if part of the capital eventually flows into the real economy, financing enterprises have to bear high financing costs under the leverage of shadow banking. Therefore, enterprises’ financing costs and financing constraints rise, and the financing dilemma intensifies, inhibiting corporate G-innovation. This lead to our fourth hypothesis:

**Hypothesis 4.** *The expansion of shadow banking will ease the inhibition of financing constraints on G-innovation*.

Based on the theoretical analysis, we establish an econometric model to empirically test the performance of G-innovation in the context of financing constraints. The specific model is as follows:

$${\mathrm{Greeninnovation}}_{\mathrm{it}}=\beta_{0}+\beta_{1}{\mathrm{FC}}_{\mathrm{it}}+\beta_{2}{\mathrm{Controls}}_{\mathrm{it}}+\varepsilon_{\mathrm{it}}$$ (1)

$${\mathrm{Greeninnovation}}_{\mathrm{it}}=\beta_{0}+\beta_{1}{\mathrm{FC}}_{\mathrm{it}}{}^{*}{\mathrm{Environment}}_{t}+\beta_{2}{\mathrm{Controls}}_{\mathrm{it}}+\varepsilon_{\mathrm{it}}$$ (2)

Among them, Greeninnovation_it_ indicates the explained variable “corporate G-innovation”, FC_it_ indicates the variable “financing constraints”, Controls_it_ indicates the control variables, and Environment_t_ indicates the environmental variable. We mainly consider two environmental variables: the economic policy uncertainty and the scale of shadow banking. We also control the firm fixed effect to exclude the influence of missing individual characteristic variables that are time invariant, and controls the year fixed effect to exclude the influence of macroeconomic environment factors.

### Variables and data

#### Variables

The explained variable is corporate G-innovation, which is indicated by the number of green invention patent applications. Because it is a discrete data with zero value, we transform it by adding to 1 and then taking the natural logarithm, ln (1+Green invention patent applications).

There is no consensus on how to measure financing constraints. The main methods include Investment-cash flow sensitivity, KZ index, WW index, SA index and so on. Fazzari, Hubbard [44] first proposed that the investment-cash flow sensitivity reveals the existence of financing constraints. Kaplan and Zingales [45] questioned their approach on the grounds that investment-cash flow sensitivity did not increase monotonously with the increase of financing constraints, and investment opportunities may not be fully controlled. The actual KZ index comes from the research of Lamont, Polk [46], who use an ordered logit model to connect the degree of financing constraints with five accounting variables: cash flow, P/E ratio, leverage ratio, dividend and cash holdings, and use the estimated regression coefficient to construct the KZ index. The higher the index value is, the greater the financing constraints on enterprises. Hadlock and Pierce [47] updated the KZ index’s text-based method by searching the financial statements of 356 companies randomly selected from 1995 to 2004 to identify which companies are subject to financing constraints. Using this classification, they found that financing constraints index can be constructed based on size, square of size and age. Like the KZ index, the subsequent users of HP index calculate the financing constraint index by applying the coefficient of SA index to their own samples. Based on the coefficient of structural model, Whited and Wu [48] constructed an index which was the weighted result of the following variables: cash flow of assets, whether to pay dividends, the ratio of long-term liabilities to total assets, scale, sales growth and industry sales growth. Subsequent users called it the WW index. Furthermore, Denis and Sibilkov [49] and Lee and Park [50] thought that there were more or less applicability problems when using SA index, KZ index and WW index, and they selected four variables “enterprise size, annual payout ratio, bond rating and paper rating” to measure financing constraints. Therefore, in consideration of China’s actual situation, we select six variables such as SA index, KZ index, WW index, firm size, firm’s establishment duration and dividend per share to measure financing constraints. The specific calculation process is shown in S1 Appendix.

According to relevant theories about the factors affecting corporate technological innovation, we select the following control variables:

1. Ownership structure. The relationship between ownership concentration and technology innovation is still controversial. On the one hand, some scholars argue that improving ownership concentration may be conducive to technological innovation.Shleifer and Vishny [51] argue that the increase of shareholding ratio will strengthen major shareholders’ supervision on manager’s opportunistic behavior and promote technological innovation. On the other hand, some scholars argue that the improvement of ownership concentration hinders technological innovation. Demsetz and Lehn [52] argue that when the shareholding ratio is high enough, large shareholders have the ability to encroach the interests of the small shareholders by controlling the decision-making of the company, which is generally known as the "expropriation effect" of the large shareholders.
2. The holding level of institutional investors. It is generally believed that improving the level of institutional investors can promote technological innovation. According to the research of Aghion, Van Reenen [53], when institutional investors hold a high proportion of shares, professional managers will pay attention to improving the level of technological innovation in order to avoid personal professional risk.
3. Enterprise profitability. R&D projects have long duration and high uncertainty, which makes it difficult for enterprises to attract external investment in R&D projects [54]. Enterprises need to have certain internal financial resources to support R&D projects, so profitability is very important for corporate innovation [55]. Audretsch [56] argue that firms with higher profitability are more willing to carry out technological innovation.

Other explanatory variables are shown in **Table 1**. Because the application of green invention patent often takes a certain period of time, the explanatory variables are taken their one year lag value.

**Table 1:** Main variables and their measurement methods.

Variable type	Variable name	Measurement methods
Explained Variable	Corporate G-innovation	Ln(1+ Number of green invention patent applications)
Explanatory Variable	Financing constraints	As shown in the appendix A
	Profitability	Net profit divided by assets
	Ownership structure	A-share ratio of top 10 shareholders
	institutional investors	the A-share ratio of institutional investors
Group variables: enterprise attribute variables	Ownership nature	Whether it is a state-owned enterprise or not
	Different political connections	When the key executive, chairman or general manager of a listed company is (or has been) a government official, a deputy to the National People’s Congress or a member of the Chinese people’s Political Consultative Conference, the variable is 1; otherwise, is 0
	Enterprises in different industries	When the enterprise is in heavy pollution industry, the variable is 1, otherwise is 0
Group variables: environment variables	Development of shadow banking	The scale of shadow banking
	Economic policy uncertainty	EPU index

#### Data

The samples are mainly Chinese A-share listed companies over the period 2007–2019, and the data is mainly collected from CSMAR database. We select the time starting point at 2007 in consideration of the revision of “China’s accounting standards for business enterprises” in 2006. According the research of Hong, Drakeford [57],we collect the data of invention patents from the website of the State Intellectual Property Office in China, and identify the green invention patents according to the IPC Green Inventory, which is an online tool launched by the World Intellectual Property Organization (WIPO).

Considering the comparability of samples, we mainly make the following data cleaning: (1) We exclude financial enterprises and ST (Special Treatment) enterprises for the sake of comparability. Specially, the accounting system of financial enterprises is quite different, and ST enterprises have abnormal operating conditions, which make them quite different from other enterprises and not suitable for our research, so these observations should be dropped. (2) We drop observations with missing values.(3) We drop observations that are obviously unreasonable, such as ones with asset liability ratio greater than 100%.Finally, we get 18570 firm-year observations. In order to compare the differences of invention patent applications under different financing constraints, we classify the samples into three groups such as severe financing constraints, moderate financing constraints and low financing constraints and then make a descriptive analysis. The results are shown in **Table 2**.

**Table 2:** Grouped descriptive statistical results of green invention patent applications.

Variable	Obs	Mean	Std.Dev.	Min	max
Large KZ	6189	0.282	0.711	0	5.964
Medium KZ	6191	0.300	0.730	0	6.941
Small KZ	6190	0.290	0.710	0	7.239
Large SA	6189	0.255	0.620	0	6.850
Medium SA	6192	0.292	0.696	0	7.239
Small SA	6189	0.325	0.819	0	7.150
Large WW	6190	0.168	0.466	0	4.043
Medium WW	6190	0.261	0.616	0	5.468
Small WW	6190	0.444	0.952	0	7.239
Large Size	6190	0.441	0.952	0	7.239
Medium Size	6190	0.249	0.603	0	4.174
Small Size	6190	0.182	0.485	0	5.468
Large Age	6191	0.265	0.715	0	7.150
Medium Age	6190	0.315	0.749	0	7.239
Small Age	6189	0.291	0.684	0	6.850
Large Dividend	6192	0.368	0.846	0	7.239
Medium Dividend	6189	0.295	0.688	0	5.784
Small Dividend	6189	0.210	0.584	0	5.894

According to **Table 2**, there are fewer green invention patent applications in severe financing constraints groups, than those in moderate financing constraints groups and low financing constraints groups. Specifically, in the four groups of SA index, WW index, Size and Dividend, firms with low financing constraints on average have applied the most green invention patents, followed by those with moderate financing constraints, and those with severe financing constraints. In the other two groups of Age and KZ index, firms with severe financing constraints have applied for the fewest green invention patents, followed by those with low financing constraints, and firms with moderate financing constraints, have applied for the most green invention patents.

Before the regression analysis, we analyze the correlation coefficient of the six financial constraint indexes to judge whether there is a high consistency within these indexes. **Table 3** shows the results of Pearson correlation coefficient matrix.

**Table 3:** The correlation coefficient matrix of financial constraint indexes.

	SA	WW	KZ	SIZE	AGE	DIV
SA	1					
WW	0.4812*	1				
KZ	-0.2491*	0.0103	1			
SIZE	0.6222*	0.8272*	-0.2103	1		
AGE	0.8106*	0.1321*	-0.1557*	0.2045*	1	
DIV	0.0467*	0.2330*	0.4789*	0.1553*	-0.0115	1

Notes:

* indicate significant at the levels of 1%.

According to **Table 3**, there is high consistency among the indicators about financing constraints, indicating a high credibility of these indicators. Specifically, there is a significant positive correlation between SA index, WW index, Size and Dividend, indicating a high degree of consistency. Age has a significant positive correlation with SA Index, WW Index and Size, While KZ index has a significant negative correlation with SA index and Age, indicating a poor consistency, and thus a poor credibility of KZ Index. But KZ index is still widely used in practice, so in our analysis, we still take KZ Index as one of the methods to measure financing constraints.

### Corporate G-innovation behavior under the background of financing constraints

Considering that existing research has not arrived at a conclusion on how to measure financing constraints, we uses six indicators to measure the financing constraints in the empirical analysis, and adds four control variables into the model, such as the proportion of state-owned shares (*stateown*), the proportion of institutional investors (*ins*), the ratio of net profits to assets (*roa*) reflecting profitability, and the proportion of top ten shareholders (*topten*) reflecting the ownership structure. Results is shown in **Table 4**.

**Table 4:** The impact of financing constraints on corporate G-innovation.

	(1)	(2)	(3)	(4)	(5)	(6)
	KZ	WW	SA	Size	Age	DIV
FC	-0.0053	-0.2895***	-0.5279***	-0.0644***	-0.0275***	-0.1102***
	(-1.10)	(-2.80)	(-7.55)	(-6.26)	(-13.01)	(-4.16)
stateown	0.0371	0.0332	0.0164	0.0260	0.0364	0.0368
	(1.01)	(0.90)	(0.45)	(0.71)	(0.99)	(1.00)
ins	0.0208	0.0196	0.0246	0.0258	0.0204	0.0185
	(0.77)	(0.73)	(0.92)	(0.96)	(0.76)	(0.69)
roa	0.2241**	0.2033**	0.2726***	0.2747***	0.2559***	0.1671*
	(2.28)	(2.13)	(2.92)	(2.93)	(2.73)	(1.74)
topten	-0.0022***	-0.0023***	-0.0026***	-0.0026***	-0.0021***	-0.0024***
	(-3.99)	(-4.20)	(-4.70)	(-4.76)	(-3.84)	(-4.30)
Firm FE	Yes	Yes	Yes	Yes	Yes	Yes
Year FE	Yes	Yes	Yes	Yes	Yes	Yes
*N*	14920	14920	14920	14920	14920	14920
*R* ^2^	0.041	0.041	0.045	0.044	0.041	0.042

Notes: T values in brackets

*, **, ***, respectively indicate significant at the levels of 10%, 5% and 1%; FC in columns (1)—(6) indicates KZ index, WW index, SA index, opposite number of enterprise scale, opposite number of enterprise establishment duration and opposite number of dividend per share, respectively.

It can be concluded from **Table 4** that the increase of financing constraints will force firms to reduce the applications of green invention patent. Specifically, from column (2) to column (6), the coefficients of the variable FC are significantly negative, which indicates that firms will choose to reduce the applications of green invention patent when financing constraints increase. In column (1), the coefficient of financing constraints is also negative, but not significant (t value is—1.1). This does not mean that financing constraints don’t have inhibitory effect on G-innovation. Because every index has its own limitations with no exception for KZ index, for example, Almeida, Campello [58] argue that KZ index is not suitable to discriminate financing constrained enterprises from non-financing constrained enterprises through the change of cash policies, while in the study of Schauer, Elsas [59], they argue that the KZ index have a higher discrimination power than SA index. Therefore, it is better to comprehensively examine the impact of financing constraints through multiple indicators.

In summary, the existing research on financing constraints and enterprise technological innovation is controversial. Some studies believe that more money can bring more technological innovation, so they believe that financing constraints will hinder enterprise technological innovation; Some studies believe that less money can bring better technological innovation. They believe that financing constraints will encourage enterprises to choose more innovative projects. Our results support the first view that financing constraints reduce the number of green invention patent applications. The possible reason lies in that the social benefits of green technology innovation are greater than personal benefits, which makes enterprises tend to reduce the R & D investment of green technology innovation when facing financing constraints.

In addition, we also find that the coefficient of the variable “*topten*” is negative and significant at the 1% level, which indicates that the higher the proportion of the top ten shareholders, the lower the level of G-innovation. It supports the hypothesis of "encroachment effect" of large shareholders, that is, when the ownership concentration increases, large shareholders may be able to collude with managers to encroach the interests of small shareholders. The coefficient of the variable “*roa*” is significantly positive, indicating that the stronger the profitability of enterprises, the higher G-innovation enterprises exhibit. However, neither the variable “*stateown*” nor “*ins*” is significant, which indicates that increasing the proportion of state-owned shares and institutional investors does not affect corporate green technological innovation.

## The discretionary effort on G-innovation of enterprises with different attributes

Firm’s attributes mainly include property rights, pollution degrees and political connections, which may play an important role in G-innovation. And enterprises with different attributes may be faced with different financing constraints. We further distinguish enterprises with different attributes and studies their G-innovation behavior respectively.

### Based on different property rights

First of all, according to the nature of property rights, we divide enterprises into state-owned enterprises and non-state-owned enterprises, and make regression analysis respectively. The results are shown in **Table 5**.

**Table 5:** Heterogeneous G-innovation behavior of enterprises with different property rights.

	(1)	(2)	(3)	(4)	(5)	(6)	(7)	(8)	(9)	(10)	(11)	(12)
	State-owned enterprises						Non-State-owned enterprises					
SA	-0.92***						-0.40***					
	(-8.05)						(-4.24)					
WW		-0.12						-0.48***				
		(-0.76)						(-3.26)				
KZ			-0.00						-0.00			
			(-0.42)						(-0.77)			
Size				-0.09***						-0.07***		
				(5.14)						(4.76)		
Age					-0.03***						-0.02***	
					(10.41)						(7.11)	
Div						-0.09***						-0.12***
						(2.92)						(2.78)
*N*	6546	6546	6546	6546	6546	6546	8374	8374	8374	8374	8374	8374
*R* ^2^	0.063	0.052	0.052	0.057	0.052	0.054	0.036	0.035	0.034	0.037	0.034	0.035

Notes: T values in brackets

*, **, ***, respectively indicate that they are significant at the levels of 10%, 5% and 1%. Other control variables are added to the model, and the year fixed effect and firm fixed effect are controlled. Column (1)—column (6) shows the impact of financing constraints on G-innovation of state-owned enterprises; column (7)—column (12) shows the impact of financing constraints on G-innovation of non- state-owned enterprises.

It can be concluded from Table 5 that in both state-owned enterprises and non-state-owned enterprises, financing constraints will inhibit their applications of G-innovation, but there is no heterogeneity between them. Comparing columns (1)—(6) with columns (7)—(12), we find that most of the coefficients on the variables “financing constraint” are significantly negative. This shows that no matter state-owned enterprises or non-state-owned enterprises, financing constraints have a significant inhibitory effect on their G-innovation. Furthermore, we add the interaction term between state-owned enterprises and financing constraints into the model, and find that the coefficient in front of the interaction term is not significant, which indicates that the effect of financing constraints on G-innovation is not heterogeneous between state-owned enterprises and non-state-owned enterprises. This shows that both state-owned enterprises and non-state-owned enterprises tend to reduce the level of green technology innovation when facing financing constraints. This situation has not been improved due to the fact that state-owned enterprises are easier to obtain financing from commercial banks.

### Based on different political connections

Then, according to the criterion "whether the chairman, general manager or the key executives of listed companies is (or has been) a government official, a deputy to the National People’s Congress, or a member of the Chinese people’s Political Consultative Conference", we divide the samples into two sub-samples: enterprises with political connections and enterprises without political connections, and make regression analysis respectively. The results are shown in **Table 6**.

**Table 6:** Heterogeneous G-innovation behavior of enterprises with different political connections.

	(1)	(2)	(3)	(4)	(5)	(6)	(7)	(8)	(9)	(10)	(11)	(12)
	Political connected enterprises						Non-political connected enterprises					
SA	-0.42**						-0.56***					
	(-2.01)						(-6.26)					
WW		-0.25						-0.32***				
		(-0.87)						(-2.63)				
KZ			0.01						-0.01**			
			(0.83)						(-2.31)			
Size				-0.04						-0.07***		
				(1.30)						(5.38)		
Age					-0.02**						-0.03***	
					(2.53)						(10.09)	
Div						-0.06						0.14***
						(-0.68)						(4.18)
*N*	4101	4101	4101	4101	4101	4101	10819	10819	10819	10819	10819	10819
*R* ^2^	0.025	0.023	0.023	0.023	0.022	0.023	0.036	0.032	0.032	0.035	0.032	0.033

Notes: T values in brackets

*, **, ***, respectively indicate that they are significant at the levels of 10%, 5% and 1%. Other control variables are also added to the model, and the year fixed effect and firm fixed effect are controlled. Column (1)—column (6) shows the impact of financing constraints on G-innovation of politically connected enterprises; column (7)—column (12) shows the impact of financing constraints on G-innovation of non- political connected enterprises.

It can be concluded from **Table 6** that enterprises with different political connections exhibit heterogeneous G-innovation behaviors. When financing constraints increase, enterprises with non-political connections will significantly reduce the applications of green invention patent. Comparing column (1)—(6) with column (7)—(12), we find that in the subsamples of politically connected enterprises, only the coefficients of the SA index and Age variable are significantly negative, while in the subsamples of non-political connected enterprises, all the six proxy indexes are significantly negative. This indicates that the increase of financing constraints will significantly inhibit non-political enterprises from applying for green invention patent. It may be due to the fact that China’s commercial banks are mainly state-owned in nature, and enterprises with political connections can get loans from banks more easily through government officials. As a result, non-political enterprises are more vulnerable to financing constraints, and are more inclined to reduce the level of green technology innovation.

### Based on different polluting level

Furthermore, according to the industries the enterprises belong to, we divide the samples into subsamples of heavy polluting enterprises and non-heavy polluting, and then conduct regression analysis respectively. Specifically, six major industries (including thermal power, iron and steel, petrochemical, cement, nonferrous metals and chemical industry, as specified in “the 12th Five Year Plan for prevention and control of air pollution in key areas” approved by the State Council of China) are identified as heavy pollution industries[60], while other industries are defined as non-heavy pollution industries. According to “the classification management name of environmental protection inspection industry of listed companies” issued by China’s Ministry of environmental protection in 2008 and “the industry classification guidelines of listed companies” issued by China Securities Regulatory Commission in 2012, we finally define fifteen industries as heavy pollution industries, and the other as non-heavy pollution industries.

It can be concluded from **Table 7** that under the background of financing constraints, both heavy polluting enterprises and non-heavy polluting enterprises have significantly reduced the applications of green invention patents, and there is no heterogeneity between them. Comparing columns (1)—(6) with columns (7)—(12), for the sub-samples of heavy pollution enterprises, most of the coefficients on the proxy variable of financial constraint are significantly negative, which indicates that when the financing constraint increases, heavy pollution enterprises will reduce the applications of green invention patent. Similarly, for the sub-samples of non-heavy pollution enterprises, the coefficients on the proxy variable of financial constraint are basically negative, which indicates that when the financing constraints increase, non-heavy polluting enterprises will also reduce the applications of green invention patent. Furthermore, we also make an empirical test on the differences between these two sub-samples. Two variables including the interaction between the dummy variable “whether enterprises are heavy polluting ones or not” and financing constraints are added into the model. The coefficient on the interaction is not significant, which further prove the above conclusion that enterprises will reduce the applications of green invention patent whether they are heavy polluting enterprises or not, and there are no differences between these two sub-samples. And heavy polluting enterprises are more vulnerable to financing constraints, but when facing financial constraints, these two groups of enterprise will reduce their green technology innovation.

**Table 7:** Heterogeneous G-innovation behavior of enterprises with different pollution levels.

	(1)	(2)	(3)	(4)	(5)	(6)	(7)	(8)	(9)	(10)	(11)	(12)
	Heavy polluting enterprises						Non heavy polluting enterprises					
SA	-0.67***						-0.43***					
	(-5.61)						(-4.87)					
WW		-0.09						-0.38***				
		(-0.46)						(-3.05)				
KZ			0.00						-0.01			
			(0.36)						(-1.35)			
Size				-0.07***						-0.07***		
				(-3.56)						(-5.37)		
Age					-0.03***						-0.03***	
					(-7.63)						(-10.63)	
Div						-0.04						-0.21***
						(-1.26)						(-4.86)
*N*	5833	5833	5833	5833	5833	5833	9087	9087	9087	9087	9087	9087
*R* ^2^	0.058	0.052	0.052	0.054	0.052	0.052	0.039	0.038	0.037	0.040	0.036	0.039

Notes: T values in brackets

*, **, ***, respectively indicate that they are significant at the levels of 10%, 5% and 1%. Other control variables are added to the model, and the year fixed effect and firm fixed effect are controlled. Column (1)—column (6) shows the impact of financing constraints on G-innovation of heavy polluting enterprises; column (7)—column (12) shows the impact of financing constraints on G-innovation of non- heavy polluting enterprises.

## The discretionary effort on G-innovation of enterprises with different external environment

### Based on economic policy uncertainty

This paper mainly uses the EPU index constructed by Baker, Bloom [61] to measure the economic policy uncertainty. The index is constructed through the text analysis of uncertainty based on the daily news content of the South China Morning Post. It have several advantages so that it has been widely cited [62–64], for example, it directly depicts the economic policy uncertainty as a whole, and it is consistent with the construction of relevant indexes in the United States and other countries. Therefore, on the basis of the above model, we add two variables such as economic policy uncertainty index and its interaction with financing constraints to analyze how economic policy uncertainty affects corporate G-innovation behavior when financing constraints increase. The results are shown in **Table 8**.

**Table 8:** The impact of economic policy uncertainty on corporate G-innovation behavior.

	(1)	(2)	(3)	(4)	(5)	(6)
SA	-0.430***					
	(-5.22)					
SA*EPU	-0.001**					
	(-2.26)					
WW		0.894***				
		(4.17)				
WW*EPU		-0.009***				
		(-6.31)				
KZZ			-0.001			
			(-0.05)			
KZZ*EPU			-0.000			
			(-0.43)			
Size				0.003		
				(0.19)		
Size*EPU				-0.001***		
				(-6.35)		
Age					-0.036***	
					(-5.14)	
Age*EPU					0.000	
					(1.29)	
Div						0.001
						(0.02)
Div*EPU						-0.001**
						(-1.96)
*N*	14920	14920	14920	14920	14920	14920
*R* ^2^	0.046	0.044	0.041	0.042	0.041	0.047

Notes: T values in brackets

*, **, ***, respectively indicate that they are significant at the levels of 10%, 5% and 1%. Other control variables are added to the model, and the year fixed effect and firm fixed effect are controlled.

It can be concluded from **Table 8** that the economic policy uncertainty will affect the firms’ G-innovation behavior. When the economic policy uncertainty increases, the inhibition effect of financing constraints on G-innovation is greater. Specifically, the coefficients on the interaction of financing constraints and economic policy uncertainty index in columns (1)—(6) are significantly negative, indicating that when the economic policy uncertainty increases, enterprises will lower the level of G-innovation to a greater extent. In other words, when economic policy uncertainty increases, enterprises tend to reduce their green technology innovation in the face of uncertainty, so as to avoid the resulting risks.

### Based on shadow banking development

We also consider the role of shadow banking development as an important external environmental factor. Specifically, in the study, we add two variables such as the scale of shadow banking and its interaction with the financing constraint to analyze how the external environment of shadow banking development affects corporate G-innovation choice behavior when the financing constraint increases. The results are shown in Table 9. In China, the broad sense of shadow banking includes: bank financial products, entrusted loans, trust loans, P2P and various private loans. This paper mainly uses the definition of core shadow banking in “China’s quarterly shadow banking monitoring report” issued by Moody. We indicate the scale of shadow banking every year by the sum of trust loan, entrusted loan and undiscounted bank acceptance balance in the stock of social financing scale.

**Table 9:** The impact of shadow banking development on corporate G-innovation behavior.

	(1)	(2)	(3)	(4)	(5)	(6)
SA	-0.435***					
	(-5.67)					
SA*shadow	-0.001***					
	(-2.97)					
WW		1.094***				
		(6.80)				
WW*shadow		-0.007***				
		(-11.23)				
KZZ			0.003			
			(0.33)			
KZZ*shadow			-0.000			
			(-1.16)			
Size				0.027**		
				(2.12)		
Size*shadow				-0.000***		
				(-12.21)		
Age					-0.047***	
					(-6.76)	
Age*shadow					0.000***	
					(3.01)	
Div						0.175***
						(2.77)
Div*shadow						-0.001***
						(-4.98)
*N*	14920	14920	14920	14920	14920	14920
*R* ^2^	0.046	0.051	0.041	0.055	0.041	0.044

Note: T values in brackets

*, **, ***, respectively indicate that they are significant at the levels of 10%, 5% and 1%. Other control variables are also added to the model, and the year fixed effect and firm fixed effect are controlled.

It can be concluded from **Table 9** that the development of shadow banking will also affect corporate G-innovation behavior, but the development of shadow banking fails to alleviate the inhibition of financing constraints on G-innovation, instead increases the inhibition of financing constraints on G-innovation. The possible explanation lies in: on the one hand, in the process of financial marketization, the expansion of the shadow banking has raised corporate financing cost for technological innovation; on the other hand, the development of shadow banking has a crowding out effect on traditional commercial banking business, which will aggravate the financing constraints of enterprises. Under the influence of these two factors, the development of shadow banking has failed to alleviate the inhibitory impact of financing constraints on corporate G-innovation.

## Conclusions

We mainly examine corporate discretionary effort on G-innovation with financing constraints. Based on the theoretical analysis, using the data of Chinese A-share listed companies from 2007 to 2019, we manually collected data on green invention patents from the website of the State Intellectual Property Office in China, and identify the green invention patents according to the IPC Green Inventory. And then we empirically analyze how financing constraints affect firms’ efforts on corporate G-innovation and compare the differences among firms with different attributes, and under different external environment. We conclude that:

Firstly, enterprises will reduce the application of green invention patents when faced with more financing constraints. This is because, differ from physical investment, R&D investment has no lateral value, no fixed cash flow, but has high risk. These characters lead to the fact that banks are not willing to lend to enterprises for R&D investment, while China’s financial structure is dominated by bank credit. Final results are that when faced with financing constraints, enterprises have to reduce G-innovation.

Then, enterprises with different attributes show heterogeneous G-innovation behavior, which is mainly reflected in enterprises with different political connections. With the increase of financing constraints, non-political connected enterprises have reduced the green invent patents sharply. However, the property structure and the pollution degree have no effect on the willingness of G-innovation. This is because enterprises closely connected with the central or local government are easier to obtain funds from the state-owned commercial banks, which makes them carry out R&D investment activities with less consideration for financing constraints.

Finally, enterprises also exhibit heterogeneous G-innovation behavior under different external environment conditions. When economic policy uncertainty increases, the inhibition of financing constraints on G-innovation become more severe, and enterprises are more reluctant to apply for green invention patents. This is mainly due to the irreversibility of R&D investment. The economic policy uncertainty will often lead to preventive delay for investment, especially for R&D investment and thus reduce the level of G-innovation. However, although the scale of shadow banking has been expanding and can make up for the lack of formal credit to a large extent, the inhibition of financing constraints on G-innovation has not been alleviated by the expansion of shadow banking. The reason is twofold. On the one hand, the expansion of shadow banking reflects the difficulty for enterprises to obtain bank loans from formal channels. On the other hand, it will raise firms’ financing cost and worsen the financing constraints.

Our research adds to the literature about the relationship between financing constraints and corporate technology innovation. It supports the view that financing constraints can hinder enterprise technological innovation. And we also make an explanation that the social benefits of green technology innovation are greater than personal benefits, which makes enterprises tend to reduce the R&D investment of green technology innovation when facing financing constraints.

But this research also has several limitations, one of which is that although the invention patents can reflect corporate green technology innovation to a certain extent, perhaps only by combining corporate R&D expenses, can we better understand corporate technology innovation behavior. Further identifying the motivation of green technology innovation is also the future research direction.

## Supporting information

S1 Data (XLSX)

S1 Appendix (DOCX)
