# Supplementary material for: Discretionary Effort on Green Technology Innovation: How Chinese Enterprises Act when Facing Financing Constraints
Source: PLoS One. 2021 Dec 22;16(12):e0261589. doi: 10.1371/journal.pone.0261589 (PMC8694420; doi:10.1371/journal.pone.0261589)
Supplement: S1 Appendix — (DOCX) [file pone.0261589.s002.docx]

# Appendix A

The Calculation of financing constraints:

①SA. It is calculated as -0.737*Size+0.043*Size2-0.04*Age. Size is indicated by the natural logarithm of the total assets at the end of the year (in millions of yuan), and Age is indicated by the establishment duration of the enterprises (in years).

②WW. It is calculated as -0.091*Cashflow-0.062*Isdivdiend+0.021*LongLiabilities-0.044*Size +0.102*IndustryIncome-0.035*Incomerate. Cashflow is indicated by Net cash flow from operating activities over Total assets at the end of the year. IsDivdiend is indicated by Whether the firm has paid cash dividends at this year or not. LongLiabilities is indicated by the ratio of long term liabilities to total assets. Size is indicated by the natural logarithm of the total assets at the end of the year (in millions of yuan). IndustryIncome is indicated by Industry sales growth rate. Incomerate is indicated by Growth rate of sales revenue.

③KZ. It is calculated as -1.002*Cashflow+0.283*Q+3.139*DebtRatio-39.368*Divdiend-1.315*Cash. Cashflow is indicated by Net cash flow from operating activities over Total assets at the end of the year. Q is indicated by Tobin Q. DebtRatio is indicated by the ratio of liability to asset at the end of the year. Divdiend .It is indicated by Cash dividends over total assets at the beginning of the year. Cash is indicated by closing balance of cash and cash equivalents over total assets at the beginning of the year.

④Size. It is calculated as opposite number of firm size. And Size is indicated by the natural logarithm of the total assets at the end of the year (in millions of yuan).

⑤Age. It is calculated as opposite number of firm’s establishment duration.

⑥Dividend. It is calculated as opposite number of dividend per share.
